# ELIHKSIR Web Server: Evolutionary Links Inferred for Histidine Kinase Sensors Interacting with Response Regulators

**DOI:** 10.3390/e23020170

**Published:** 2021-01-30

**Authors:** Claude Sinner, Cheyenne Ziegler, Yun Ho Jung, Xianli Jiang, Faruck Morcos

**Affiliations:** 1 Department of Biological Sciences, University of Texas at Dallas, Richardson, TX 75080, USA; claude.sinner@utdallas.edu (C.S.); cheyenne.ziegler@utdallas.edu (C.Z.); yxj180001@utdallas.edu (Y.H.J.); xianli.jiang@utdallas.edu (X.J.); 2 Center for Systems Biology, University of Texas at Dallas, Richardson, TX 75080, USA; 3 Department of Bioengineering, University of Texas at Dallas, Richardson, TX 75080, USA

**Keywords:** statistical inference, mutational phenotypes, interaction specificity, phosphorylation, fitness landscape, bacterial signaling

## Abstract

Two-component systems (TCS) are signaling machinery that consist of a histidine kinases (HK) and response regulator (RR). When an environmental change is detected, the HK phosphorylates its cognate response regulator (RR). While cognate interactions were considered orthogonal, experimental evidence shows the prevalence of crosstalk interactions between non-cognate HK–RR pairs. Currently, crosstalk interactions have been demonstrated for TCS proteins in a limited number of organisms. By providing specificity predictions across entire TCS networks for a large variety of organisms, the ELIHKSIR web server assists users in identifying interactions for TCS proteins and their mutants. To generate specificity scores, a global probabilistic model was used to identify interfacial couplings and local fields from sequence information. These couplings and local fields were then used to construct Hamiltonian scores for positions with encoded specificity, resulting in the specificity score. These methods were applied to 6676 organisms available on the ELIHKSIR web server. Due to the ability to mutate proteins and display the resulting network changes, there are nearly endless combinations of TCS networks to analyze using ELIHKSIR. The functionality of ELIHKSIR allows users to perform a variety of TCS network analyses and visualizations to support TCS research efforts.

## 1. Introduction

Two-component systems (TCSs) are ubiquitous in bacteria and archaea and are the key signaling transduction machineries for sensing and responding to the environment. TCSs consist of sets of interaction signaling partners, histidine kinases (HKs) that phosphorylate their cognate response regulators (RRs). Interactions, however, are often not one-to-one. Multiple HKs can interact with multiple RRs. Identifying relevant interactions among TCS is an important task that has been addressed experimentally only for a limited number of organisms.

**Figure 1:**
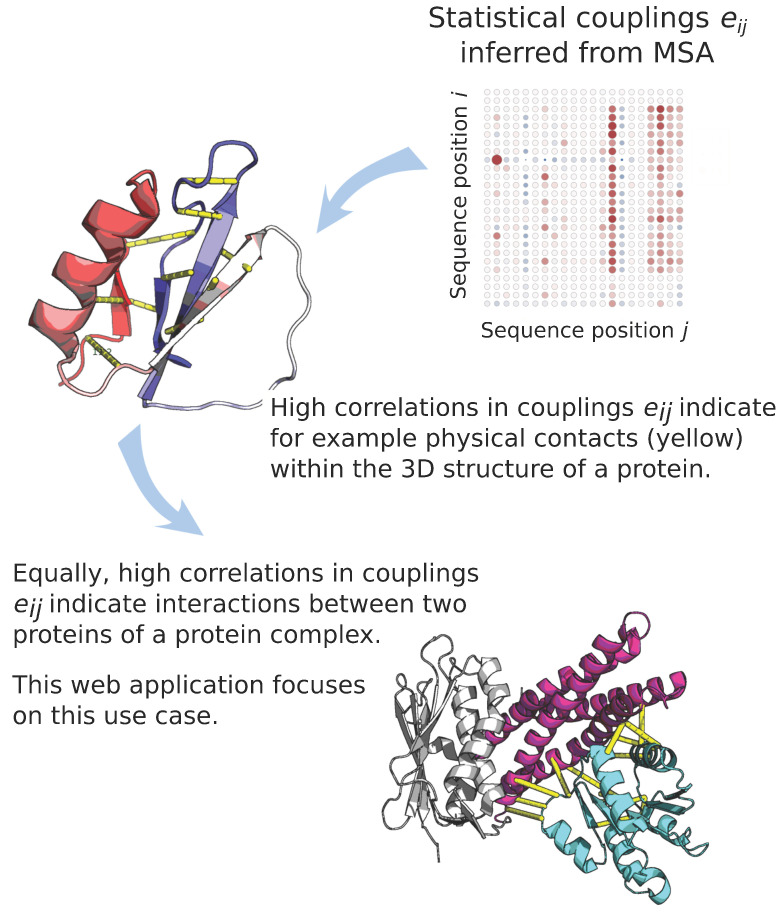
Statistical couplings for sequence position and residue type are inferred from the MSA for the protein family using the DCA method. High couplings indicate significant interactions between sequence positions. These couplings can be used to infer physical contacts within a single protein structure, or to infer the interaction interface and strength between two proteins.

We advanced the study of interaction specificity in TCS by creating a model based on amino acid coevolution at the interface of HKs and RRs. Our Direct Coupling Analysis (DCA) [1] based interaction model not only confirms known cognate partners [2] but also reveals novel interactions in multiple organisms. We uncovered a TCS network in Synechococcus elongatus regulating cyanobacterial circadian clock and confirmed important master regulators [3]. Our model is also able to predict functional mutations to modulate binding specificity between partners, such as PhoQ and PhoP [4] or even design new interactions between non-cognate, interspecies TCS proteins, such as the EnvZ from Escherichia coli and Spo0F from Bacillus subtilis [5]. Another application of this model is the identification of crosstalk across signaling networks and the influence of mutation in the topology of the network. Figure 1 illustrates a section of statistical couplings in a protein sequence and highlights two of the most common applications, the identification of physical contacts in a protein [6,7] or the identification and quantification of interactions between multiple proteins [8,9].

We decided to make this model and tools available to the scientific community in an interactive web server that facilitates the analysis and prediction of TCS networks as well as the exploration of the effects of mutation in these proteins prior to experimental work. We named the service **E**volutionary **L**inks **I**nferred for **H**istidine **K**inase **S**ensors **I**nteracting with **R**esponse regulators (ELIHKSIR) and it can be accessed at https://elihksir.org.

In recent years, online repositories of sequence data have seen a large influx of sequences and are painting a more refined picture of protein families. Using these data, one can construct global probabilistic models that verify the observed statistics and relate them to inter-residual couplings. Cheng et al. [2] have used these probabilistic models to introduce an objective function $H_{TCS}^{specific}\left(\vec{\sigma }\right)$ to describe the specificity (fitness) of the interaction between a response regulator and a histidine kinase partner by a scalar score using a sequence $\vec{\sigma }$ from a linked multiple sequence alignment (MSA). For completeness, we reproduce the introduction of $H_{TCS}^{specific}\left(\vec{\sigma }\right)$ here.

Using the set of sequences $\left\{\vec{\sigma }\right\}$, we can create a global probabilistic model $P(\vec{\sigma })$ to find a given amino acid sequence $\vec{\sigma }$ in a protein family by the following:

(1) $$P\left(\vec{\sigma }\right)=\frac{1}{Z}\cdot \exp\left(-H\left(\vec{\sigma }\right)\right)$$

with a general Hamiltonian $H(\vec{\sigma })$ and the partition function *Z* to verify normalization for the probabilities. A sufficient form for $H(\vec{\sigma })$ [10] is given by the large-q Potts Model [11]:

(2) $$H\left(\vec{\sigma }\right)\propto -\sum_{ij}e_{ij}\left(a_{i},a_{j}\right)-\sum_{i}h_{i}\left(a_{i}\right)$$

with the coupling matrix $e_{ij}\left(a_{i},a_{j}\right)$ between two sequence sites $a_{i}$, $a_{j}$ at sequence positions *i* and *j*; and the local field $h_{i}\left(a_{i}\right)$ at the site $a_{i}$ at sequence position *i*. The sites *a* can have q=21 different states for amino acid and sequence gap composition. The entries of the coupling matrix $e_{ij}\left(a_{i},a_{j}\right)$ and the local fields $h_{i}\left(a_{i}\right)$ encode preferences for sequence compositions at positions *i* and *j*. The inference of the coupling matrix $e_{ij}\left(a_{i},a_{j}\right)$ and the local fields $h_{i}\left(a_{i}\right)$ is a non-trivial task. Several methods exist to do so [1,12,13]. We inferred the couplings using mean field DCA (mfDCA), which is fast and accurate at predicting interaction specificity in TCS.

From these coupling parameters, we can introduce and create objective functions to measure varying effects. In the Material and Methods, we introduce an objective function $H_{TCS}^{specific}\left(\vec{\sigma }\right)$ that is sensitive to sequence mutations and linked to protein interaction specificity. For the calculation of $H_{TCS}^{specific}\left(\vec{\sigma }\right)$, we need full access to the couplings $e_{ij}\left(a_{i},a_{j}\right)$ and local fields $h_{i}\left(a_{i}\right)$. Throughout the process, we consider these as constant and created a database that our server uses internally to calculate new values for the $H_{TCS}^{specific}\left(\vec{\sigma }\right)$ score in a mutation event.

**Figure 2:**
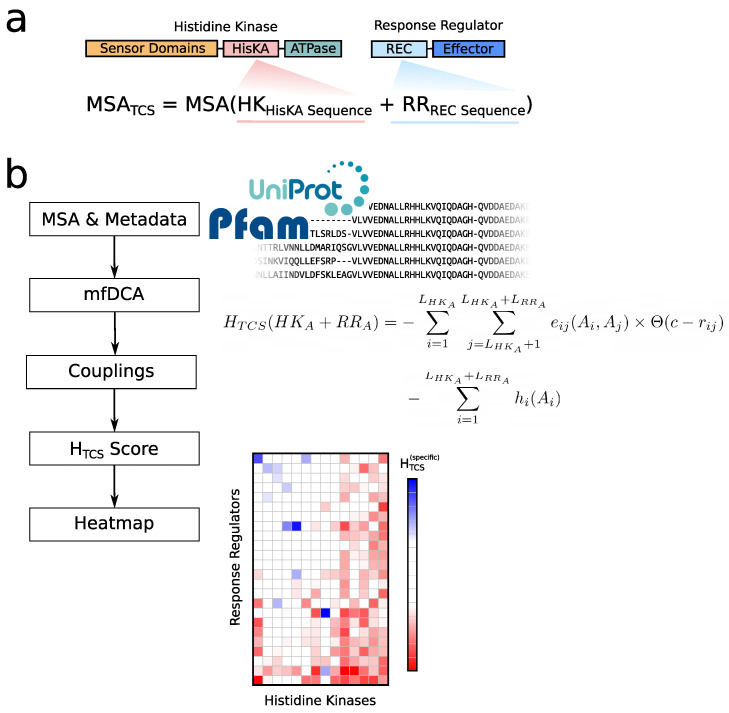
(**a**) A concatenated MSA is generated for Pfam [14] Histidine Kinase (HK) family (Pfam:PF00512) [15] and Response Regulator (RR) family (PF00072) [16]. (**b**) From this MSA coupling matrices are generated with mfDCA [1]. From these couplings, we are able to calculate a numeric score using the equation shown. This equation formally describes how Hamiltonian scores are generated for each HK–RR pair and is equivalent to Equation (3). The data are displayed in a web interface with interactive heatmaps. The user has an elaborate menu available to explore the data by creating mutations to sequence positions. The default heatmap legend is more sensitive towards the outer extremes of the values, coloring strongly negative (favorable) or positive values (unfavorable).

Figure 2 gives an overview of the entire process of the ELIHKSIR web server. The MSA for our system is created by concatenating the HisKA domain section of the Pfam [14] Histidine Kinase (HK) family (Pfam:PF00512) [15] and the REC domain of the Response Regulator (RR) family (PF00072) [16], which contains information for thousands of organisms. Furthermore, we collect metadata for each organism and sequence pairs through the Uniprot database [17]. From this, we calculate the coupling matrices $e_{ij}\left(a_{i},a_{j}\right)$ and the local fields $h_{i}\left(a_{i}\right)$. These parameters allow us to calculate a score for the interaction specificity $H_{TCS}$. The data are visualized in a web interface with interactive heatmaps.

ELIHKSIR is a user-friendly and accessible tool that displays TCS signaling networks. The breadth of the web server allows for analysis of TCS networks in both common and uncommon species and strains. Table 1 summarizes the number of organisms and interaction partners available. Users can easily search for their organism of interest, view TCS specificity networks for the whole organism, and view all possible interactions for an HK or RR of interest. This capability allows researchers with restricted computational resources to analyze signaling networks. Some common use cases of ELIHKSIR’s features include identifying cross-talk interactions between non-cognate HKs and RRs, comparing specificity of different HK–RR pairs, and comparing differences in signaling networks between species and/or strains. In addition to browsing and exporting wild-type TCS networks, mutations may be introduced into HKs and/or RRs, for which all interaction specificity scores are recalculated and displayed. This allows users to predict network-wide changes in specificity after introducing a mutation. Further applications include testing mutants for desired change(s) in specificity, guiding engineering of TCS proteins with interaction or insulation requirements, and viewing changes in specificity for new or uncommon clinical and environmental variants. With these capabilities, ELIHKSIR is an effective tool for a variety of researchers who interface with TCS proteins and signaling.

## 2. Results

### 2.1. Validation

**Figure A1:**
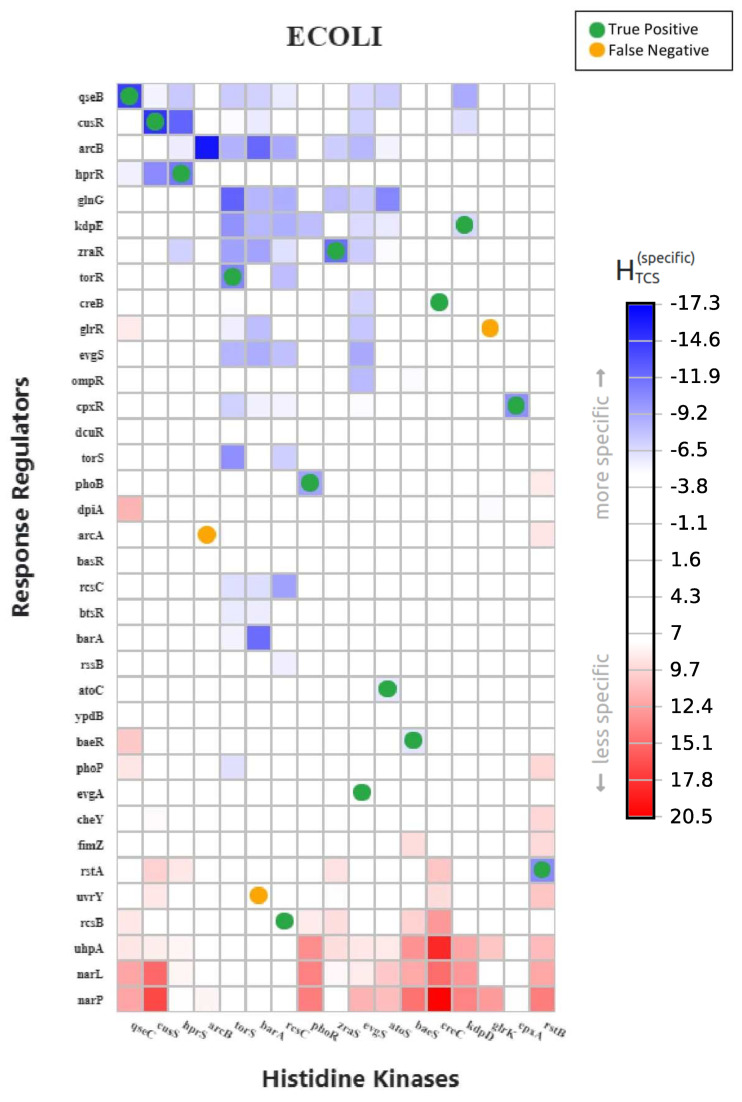
True positives are correct prediction of cognate pairs through positive and/or negative selection. False negatives occur when the cognate pairing is not the most favorable interaction.

**Figure A2:**
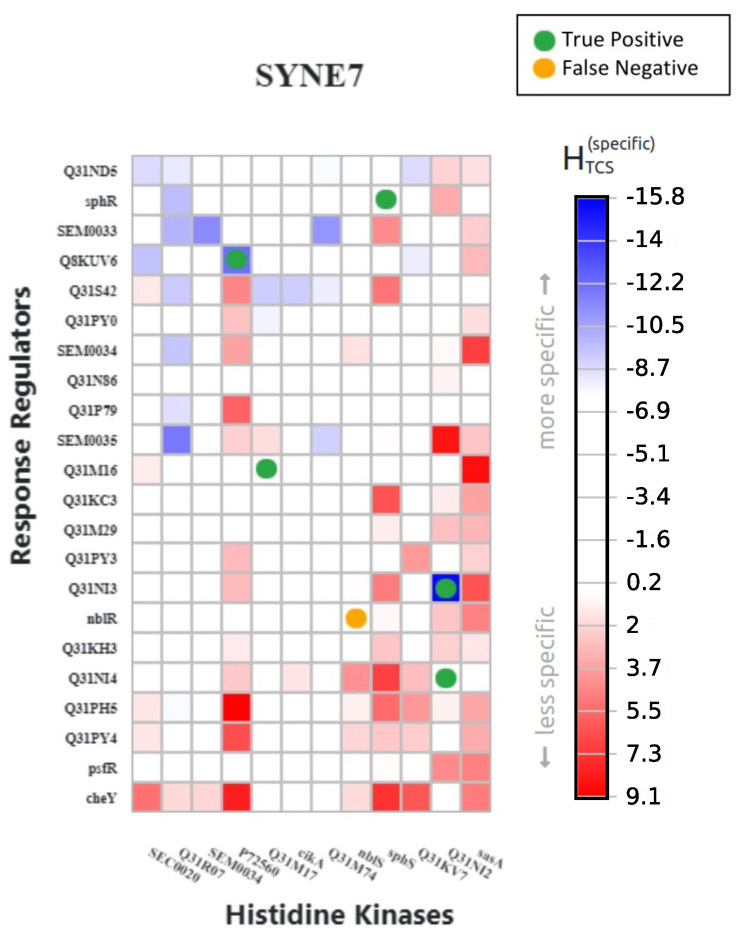
True positives are correct prediction of cognate pairs through positive and/or negative selection. False negatives occur when the cognate pairing is not the most favorable.

**Figure A3:**
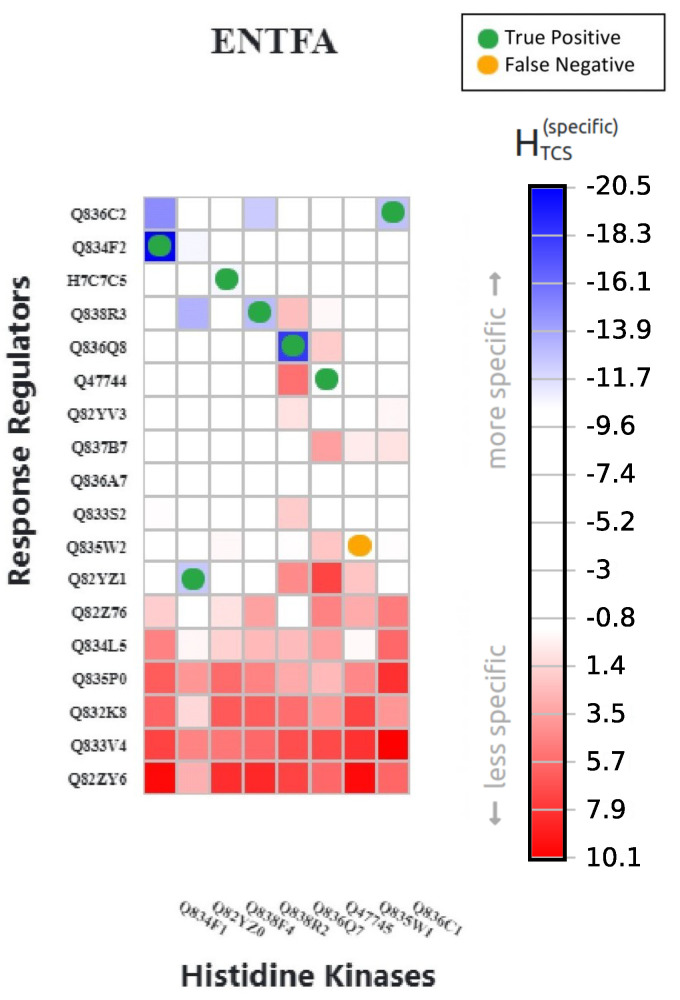
True positives are correct prediction of cognate pairs through positive and/or negative selection. False negatives occur when the cognate pairing is not the most favorable interaction.

Validation of the ELIHKSIR web server was performed through detailed investigation using three model organisms: Escherichia coli, Synechococcus elongatus, and Enterococcus faecalis. True positive specificity predictions were defined by either positive selection and/or negative selection for a cognate pair. Positive selection is defined as an HK having its highest specificity with a single RR. Negative selection is defined as an RR having poor specificity across all HKs but having its relative highest specificity with an HK. False negatives were defined as selection towards a noncognate partner that is greater than that of the cognate partner, in which both positive and negative selection fail to identify the cognate pair. Only cognate pairs in which the HK contains a HisKA domain were evaluated. For *E. coli*, there were fourteen true positives and three false negatives for seventeen cognate pairs, shown in Figure A1. For *S. elongatus*, there were five true positives and one false negative for the six cognate pairs, shown in Figure A2. For *E. faecalis*, there were seven true positives and one false negative for the eight cognate pairs, shown in Figure A3. The resulting sensitivity and accuracy is 0.84.

**Figure A4:**
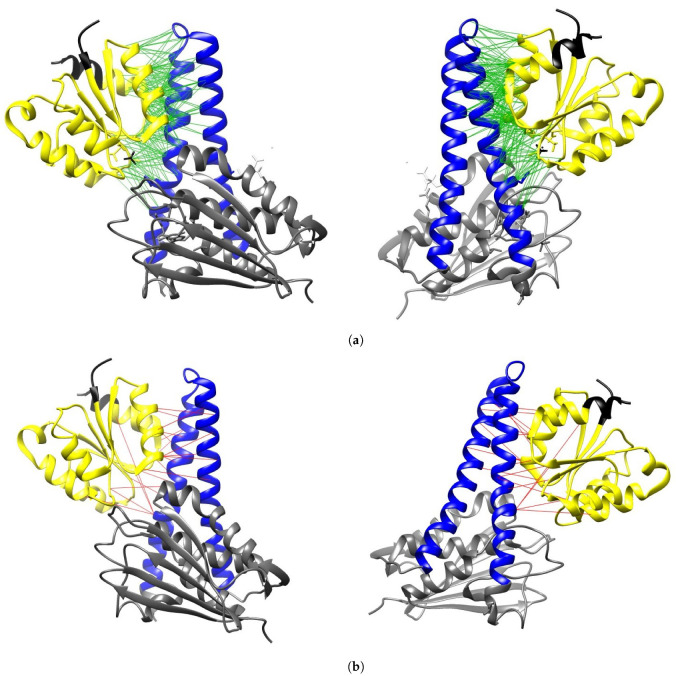
Gray structures show the HK residues lying outside of the HisKA domain. Black structures show the RR residues lying outside the REC domain. The blue structure represents the HisKA domain, and the yellow structure represents the REC domain. Green pseudobonds show contacts within 12 Angstroms C_α_ to C_α_. Red pseudobonds show the top 20 DCA couplings. The distribution of DCA couplings indicates that the model does not show biases towards subregions of the interface. (**a**) All contacts within 12 Angstroms as found in the structure viewed from two different positions, left and right faces; (**b**) Top 20 interfacial DI contacts as viewed from left and right faces.

DCA identifies coevolving residues at the HK–RR interface for HisKA and REC domains that have been used to accurately predict the structure of the HK–RR complex [18]. Out of the top 20 DCA-identified interfacial couplings, 10 are present in the 3DGE structure, as shown in Figure A4b. Information about all 3DGE interfacial contacts is present in the DCA-generated couplings and local fields (Figure A4a). Couplings are scored by their direct information (DI) value as defined by DCA (Table A2). Thus, higher DI values indicate that these couplings are more important for HK–RR interactions. When utilizing DCA couplings for the calculation of Hamiltonian values, only couplings present on the structurally verified HK–RR interface are used. This ensures auxiliary information obtained through DCA does not impact the Hamiltonian values, and thus, does not impact the resulting specificity score.

The interface is aligned for each TCS pair during the construction of the MSA, which was performed using a hidden Markov model. The sequences displayed in ELIHKSIR are the aligned residues and gaps. Predictions made based on HK and RR sequences only consider residues which align with their respective protein family. Insertions and deletions are not considered in the alignment of the interface and may result in deviations in the three dimensional structure of the resulting signaling complex. The model assumes no changes in the three dimensional structure of the HK–RR interface during evaluation of different TCS pairs.

### 2.2. Mutations

**Figure 3:**
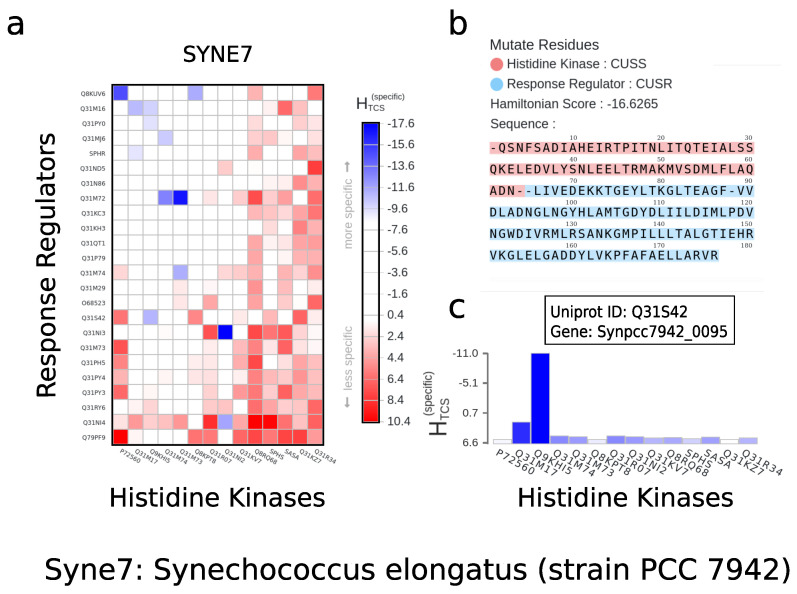
(**a**) Heatmap for Synechoccus elongatus as displayed on ELIHKSIR and when exported as an image. (**b**) Mutation screen as displayed on ELIHKSIR. (**c**) Histogram depicting all selectivity scores for a given HK or RR.

A key functionality of the ELIHKSIR server is the ability to interactively perform in silico mutations on a HK–RR pair. In the mutation screen, as shown in Figure 3b, the full MSA of a pair is shown with a visual clue to the histidine kinase region and the response regulator region. Any part of the MSA can be transformed and the changes in a HK or RR become applied globally. The heatmap is also updated accordingly. Gaps can be introduced as ’-’ characters. As the mutation values are run against a tabulated database for the positions and amino acid type, the total length of the MSA has to remain at 176 amino acids. Insertions are not possible in the model unless they occur in gap regions.

Only a subset of the positions in the genetic sequence correspond to an actual interfacial residue of the protein interface between Thermotoga maritima class I HK853 and the response regulator RR468, (PDB ID: 3DGE). Because of this, not every change in the sequence performed by a user will translate into a change in the specificity score. Furthermore, some types of amino acids can play similar roles in a specific residue position. In this case, the model accounts for this and only reflects minor or no changes in the total score.

**Figure 4:**
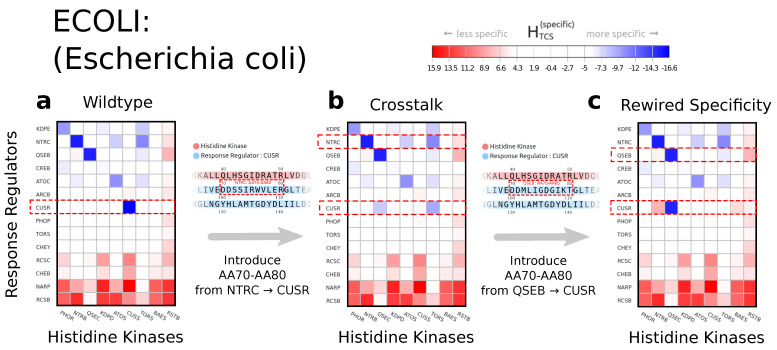
(**a**) One of the many use cases for the web server is the exploration and in silico change of specificity. In this example, we identify the response regulator cusR as the interaction partner of the histidine kinase cusS indicated by the lowest value in our Hamiltonian. (**b**) The transfer of a significant sequence portion of the response regulator ntrC does not disrupt the initial interaction and introduces cross-talk through a second interaction partner. (**c**) Alternatively, the introduction of a sequence portion of the response regulator qseB into cusR disrupts the initial interaction and rewires the interaction towards qseC.

An interesting application of the mutation user interface is shown in Figure 4, the rewiring of specificity. By transferring portions of a sequence from one cognate pair to another cognate pair, interaction properties can be discovered or lost. In this specific example, a portion of amino acids positions 70 to 80 transferred from ntrC into the same position in the cusR response regulator creates cross-talk with a new interacting partner qseC, while maintaining the initial interaction cognate partner cusS. Alternatively, introducing the same sequence positions from the response regulator qseB into cusR is entirely sufficient to rewire the entire interaction and create an exclusively positive selection towards qseC.

### 2.3. Data Export

The user has three options to export data from ELIHKSIR. First, the user may export a PNG image, as shown in Figure 3a of the entirety of the heatmap in PNG format by clicking on the Export to PNG button on the left panel once a heatmap has been displayed. This will generate a PNG image of the heatmap on a transparent background and download it onto the user’s machine. The image will also include the labels and legend. When selecting an n×m-sized subselection in a heatmap, the user is presented with the choice to display the subselection as a new heatmap. Second, the user may export a PNG image of a histogram as shown in Figure 3c of a row of response regulator and histidine kinase pairs that corresponds to a desired histidine kinase by clicking on the *Export to PNG* button that is located inside the opened histogram. The histogram export will also include the names of each response regulator. Finally, the user may export a CSV representation of the user’s arbitrary selections of the cells of the heatmap. After the user makes selections of the cells on the heatmap, the *Export to CSV* button on the right panel can be clicked to download a file that contains a comma delimited list of the user’s selections. All these methods of exporting will take into consideration the mutated Hamiltonian values, if any, of the response regulator and histidine kinase pairs.

### 2.4. Negative Selection

**Figure 5:**
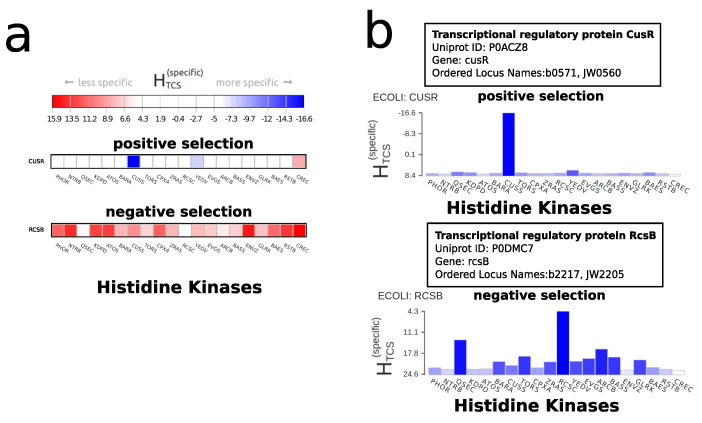
Negative selection in Escherichia coli strain K12 (ECOLI). (**a**) Heatmap view for the response regulators cusR and rcsB. In cusR, a single interaction between cusR and the histidine kinase cusS is dominant. This is a case of positive selection between two interacting partners. In rcsB, the majority of interactions are reported as having a low specificity. Even though the interaction between rcsB and the histidine kinase rcsC is not reported as having a high specificity, it will be the dominant interaction for rcsB as there is no stronger interaction partner for signal transduction. This is an example of negative selection. (**b**) Histogram view for the response regulators cusR and rcsB. From these histograms, it becomes clear that cusR-cusS (top) and rcsB-rcsC (bottom) are the dominant interactions.

An important concept highlighted by the server is that of negative selection. Not only are interaction partners indicated by strong couplings and a highly negative score for a TCS pair, but equally by high interaction scores with each partner except one. In this case, the interaction with a marginal advantage will be the strongest interaction and may facilitate signal transduction. Hence, we differentiate by either positive selection and/or negative selection for the cognate pair, where positive selection is defined as an HK having the highest specificity for its cognate RR and where negative selection is defined as the cognate HK having the highest specificity out of all HKs for a given RR. Figure 5 highlights this for two different cases in *E. coli* (ECOLI). Besides the heatmap, a good indicator for the interactions is a look at the histograms (Figure 5b) of interaction strengths, which are, for this purpose, available through the server. In cusR, a single interaction between cusR and the histidine kinase cusS is dominant (Figure 5b top). In rcsB, the majority of interactions are reported as less specific. Even though the interaction between rcsB and the histidine kinase rcsC is not reported as very specific, it will be the dominant interaction for rcsB.

## 3. Discussion

### 3.1. Characterization of Cognate Specificity

**Figure 6:**
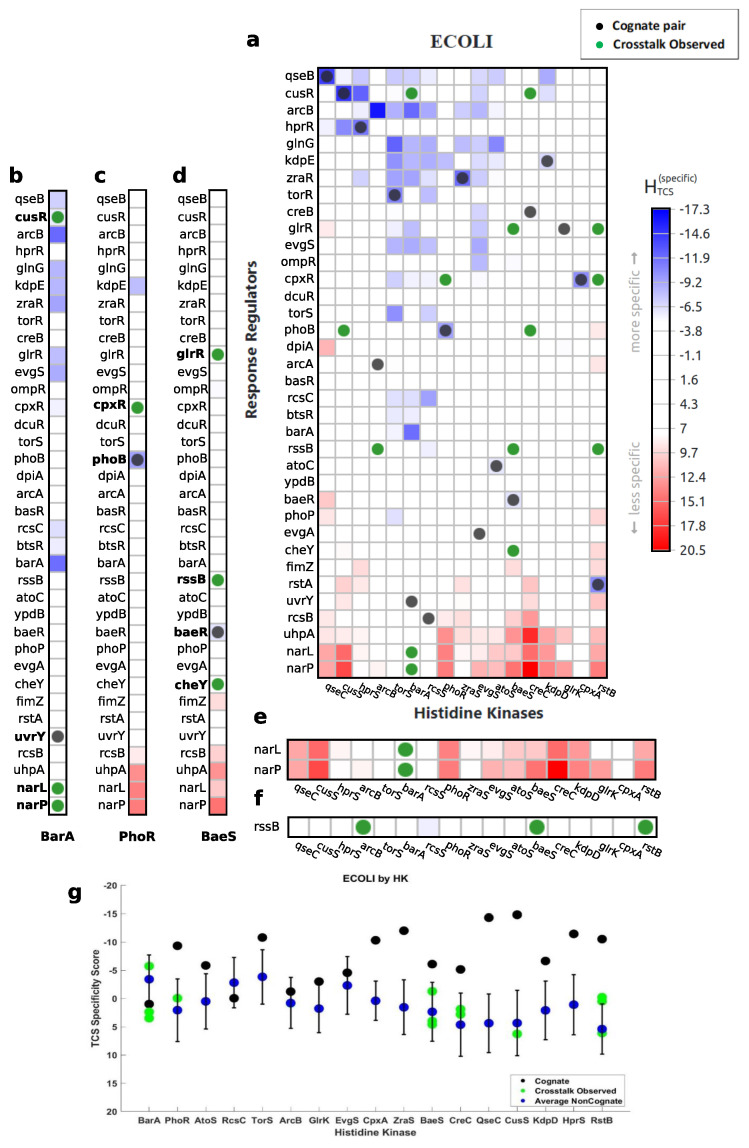
(**a**) Cognate interactions and observed in vitro crosstalk interactions overlaid onto the specificity score heatmap for *E. coli* [23]. Noncognate interactions are assessed. (**b**) BarA phosphorylates cusR, narL, and narP, in which the scores are −5.723, 2.390, and 3.491 respectively. The score for barA-cusR indicates that phosphorylation occurs due to high specificity for its noncognate partner. Phosphorylation of narL and narP are characterized in (**f**). (**c**) PhoR phosphorylates cpxR, in which the score is −0.037. A score near zero indicates diminished specificity, while still retaining attributes shared among all TCS pairs. (**d**) BaeS phosphorylates glrR, rssB, and cheY, in which the scores are −1.264, 3.998, and 4.605. The score for baeS-glrR indicates that phosphorylation occurs due to increased specificity for a noncognate partner. Phosphorylation of rssB is characterized in (**g**). Phosphorylation of cheY can be described similarly to (**f**), as its cognate HK utilizes a different family of HK than HisKA. (**e**) Cognate, crosstalk, and average non-cognate scores are shown for each HK. (**f**) HKs narQ and narX are not shown as they utilize a HisKA3 family HK, rather than HisKA. Their RRs, narL and narP, have low specificity for all HKs utilizing the HisKA domain. This leads narL and narP to be nonspecific for HisKA family HKs. Despite a lack of specificity, crosstalk is observed. (**g**) RssB is an orphan RR that can be phosphorylated by multiple HKs.

**Figure 7:**
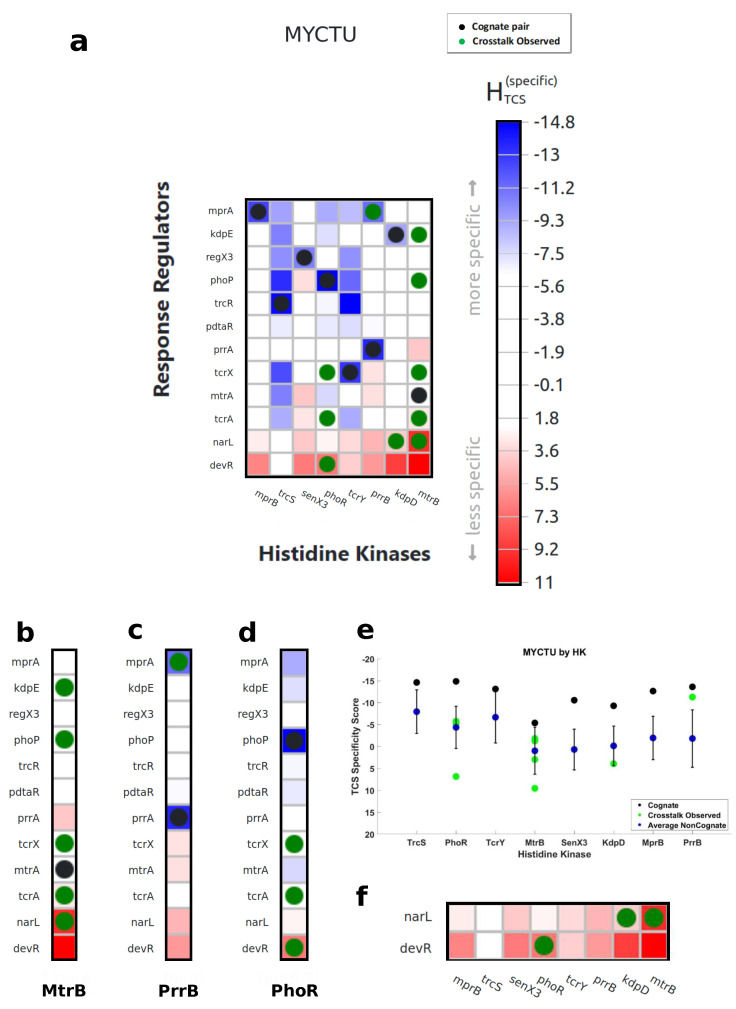
(**a**) Cognate interactions and observed in vitro crosstalk interactions overlaid onto the specificity score heatmap for M. tuberculosis [24]. Noncognate interactions are assessed. (**b**) MtrB phosphorylates kdpE, phoP, tcrX, tcrA, and narL, in which the scores are −4.895, −5.826, 0.391, −1.093, and 2.813 respectively. Scores for kdpE, phoP, and tcrA indicate that phosphorylation by mtrB occurs due to high specificity for these noncognate partners. TcrX has a score near zero, /textcolorredindicating diminished specificity but a presence of attributes shared among all TCS pairs. Phosphorylation of narL is characterized in (**f**). (**c**) PrrB phosphorylates mprA, in which the score is −11.263. This score indicates that phosphorylation of mprA by prrB occurs due to high specificity. (**d**) PhoR phosphorylates tcrX, tcrA, and devR, in which the scores are −5.744, −5.176, and 6.856, respectively. Scores for tcrX and tcrA indicate that phosphorylation by phoR occurs due to high specificity for these noncognate partners. Phosphorylation of devR is characterized in (**f**). (**e**) Cognate, crosstalk, and average noncognate scores are shown for each HK. (**f**) HKs devS and narS are not shown as they utilize a HisKA3 family HK, rather than HisKA. Their response regulators, narL and devR, have low specificity for all HKs utilizing the HisKA domain.

Through both mutational and computational analyses, the interface between the HisKA domain and the REC response regulator domain has been shown to control specificity of TCS interactions [19]. In Figure 6, this finding is confirmed for 14 out of 17 cognate pairs shown for *E. coli*. In Figure 7, this finding is confirmed for all eight cognate pairs shown for M. tuberculosis. While predictions of interaction specificity have been previously demonstrated, ELIHKSIR presents specificity scores for all HisKA HK and RR pairs in thousands of organisms, defining specificity landscapes. These specificity landscapes can then be used to determine favorable interactions through identification of pairs exhibiting positive and/or negative selection. When assessing cognate pairs, the prevalence of interactions either partially or solely characterized by negative selection becomes apparent. In the validation process, 54.8% of detected cognate pairs exhibited both positive and negative selection and 19.4% of detected cognate pairs were characterized by negative selection only. Negative selection is important for preventing cross-talk and ensuring orthogonality [20], but results indicate that it may be a main or contributing determinant of many cognate interactions. It is unclear if other attributes or domains contribute to reinforcement of specificity for cognate pairs detected by negative selection only.

By identifying whether cognate interactions are maintained by positive and/or negative selection, users can explore how deletion of TCS proteins may affect gene expression. Experimental deletion of the cognate RR in a pair regulated by negative selection may result in a noncognate RR being phosphorylated by the HK. In deletion experiments, it may be useful to understand how removal of TCS proteins may affect overall expression. Furthermore, some TCS proteins are encoded for on plasmids. Understanding how the presence or lack of plasmid-encoded TCS proteins on organisms’ genetic expression may be important for the study of antibiotic resistance and plant cell transformation by bacteria [21].

It is important to note that, in many proteins, HisKA domains are accompanied by an HATPase_c domain, which is responsible for binding ATP and transferring its γ-phosphate to the HisKA domain. Aside from its ATPase activity, the HATPase_c domain alone can act as a histidine kinase [22]. It is unknown whether the HATPase_c domain itself encodes specificity or is partially responsible for specificity in certain cognate TCS pairings. Further analysis of the HATPase_c domain as well as other histidine kinase domains could reveal additional residues and mechanisms controlling TCS orthogonality.

### 3.2. Exploration of Non-Cognate Interactions

The ELIHKSIR web server allows for exploration and visualization of signaling networks. Using the displayed heatmap, users may identify crosstalk interactions in signaling networks. Non-cognate, crosstalk interactions are common in signaling networks and may influence the expression patterns in organisms. $H_{TCS}$ scores can be used to identify non-cognate, crosstalk interactions. Non-cognate interactions may be predicted by high specificity for a non-cognate partner as shown in Figure 7b–d and Figure 6b,d. Any negative score indicates some level of encoded specificity. While scores near zero indicate no encoded specificity, TCS non-cognate partners with scores near zero may still interact due to shared attributes present in all TCS proteins, shown in Figure 6c and Figure 7b. TCS non-cognate pairs in which shared TCS attributes are partially removed have positive specificity scores, indicating low specificity. These methods of identifying possible interactions may be used across all available organisms, allowing for users to investigate crosstalk interactions within specific, and possibly uncommon, species or strains.

TCS pairs in which the RR has a cognate HK of a different family than HisKA have low specificity, but may still interact are shown in Figure 7b,d,f and Figure 6b,d,f. The ability to interact despite very low specificity indicates there may be activity of HATPase_c in phosphorylation of non-cognate RRs whose cognates belong to other HK families since HATPase_c is present in both HisKA and HisKA3 family HKs.

In Figure 6g, we observe an orphan RR that exhibits low specificity for many HKs and has been phosphorylated by HKs with low predicted specificity. Aside from the possibility of HATPase_c domain contributions, it is possible that low specificity for orphan RRs is favorable as it promotes promiscuity. In the case of rssB in *E. coli*, phosphorylation is important for function [25,26]. Therefore, promiscuity of rssB could ensure maintenance of function throughout the *E. coli* life cycle. Using similar reasoning, one can identify potential interactions with orphan HKs and RRs. Information yielded from analysis of orphan TCS proteins may assist in describing their role in organisms’ life cycles, environmental stress responses, and expression patterns. Utilizing predicted orphan TCS protein interactions could be useful in the study of antibiotic resistance in bacteria, response to environmental metals and compounds in archaea, or plant response to drought.

### 3.3. Revealing Interaction Specificity for Mutation and Variation

After mutating a protein residue, specificity scores are recalculated and the heatmap is updated. This reveals how mutation(s) change interaction specificity with all possible TCS partners. A feature that becomes important when scientists would like to assess the network effect of mutations as opposed to single pairwise interactions. The ELIHKSIR web server also separates organisms by strain, allowing interaction specificities to be compared between different strains of the same organism. Accessibility of specificity predictions for different mutants and strains may reveal differences in TCS signaling of clinical and environmental variants and may assist in the engineering of sensory kinases and response regulators as it has been shown in previous studies [5].

## 4. Materials and Methods

### 4.1. MSA Construction

Raw HMM profiles for HisKA and REC were obtained through Pfam’s hidden Markov models (HMM) [27,28]. Then, the profile was searched using Hmmer’s hmmsearch against the TrEMBL database. HKs with a sequence gap of 5 residues or larger were excluded from the MSA. The resulting HisKA domain MSA was 67 residues in length and contained 111,032 sequences utilized in the ELIHKSIR web server. RRs with a sequence gap of 6 residues or larger were excluded from the MSA. The resulting REC domain MSA was 112 residues in length and contained 225,616 sequences utilized in the ELIHKSIR web server. Cognate HK-RR pairs were concatenated and used for the generation of couplings and local fields using mfDCA, where cognate is defined by having adjacent loci [29]. The resulting cognate MSA was 179 residues in length and contained 10,091 sequences. A number of 25 iterations of random concatenation of each HK to a random RR was used to generate a scrambled MSA. The resulting MSA was 179 residues in length and contained 16,363,100 sequences.

### 4.2. mfDCA Evolutionary Couplings and Hamiltonian Scores

Mean field DCA (mfDCA) [1] was used to infer the coevolutionary parameters from conjugated multiple sequence alignments (MSAs) of cognate HK–RR sequences and scrambled HK–RR sequences. The resulting coupling parameters and local field parameters were utilized in the calculation of Hamiltonian scores. In order to quantify changes on the Hamiltonian H(S), Cheng et al. introduced a score $H_{TCS}$ as follows:

(3) $$\begin{array}{cc} H_{\mathrm{TCS}}\left({\mathrm{HK}}_{A}+{\mathrm{RR}}_{A}\right)= & -\sum_{i=1}^{L_{{\mathrm{HK}}_{A}}}\sum_{j=L_{{\mathrm{HK}}_{A}}+1}^{L_{{\mathrm{HK}}_{A}}+L_{{\mathrm{RR}}_{A}}}e_{ij}\left(A_{i},A_{j}\right)\times \Theta \left(c-r_{ij}\right)\\ & -\sum_{i=1}^{L_{{\mathrm{HK}}_{A}}+L_{{\mathrm{RR}}_{A}}}h_{i}\left(A_{i}\right) \end{array}$$

for a specific pair between a sequence ${\mathrm{HK}}_{A}$ and ${\mathrm{RR}}_{A}$ of sequence lengths $L_{{\mathrm{HK}}_{A}}$ and $L_{{\mathrm{RR}}_{A}}$ with the coupling matrix $e_{ij}\left(A_{i},A_{j}\right)$ between two sequence sites $A_{i}$, $A_{j}$ at sequence positions *i* and *j*; and the local field $h_{i}\left(A_{i}\right)$ at the site $A_{i}$ at sequence position *i*. $L_{{\mathrm{HK}}_{A}}$ is 67 for the HisKA domain and $L_{{\mathrm{RR}}_{A}}$ is 112 for the REC domain. The couplings are only taken within a pair distance $r_{ij}<c=12\;Å$ of a native contact, expressed by a function Θ(x)=1 for all x>0 and Θ(x)=0 for x≤0. The contact map of the native interfacial pairs is given by the 3D resolved structure of protein interface Thermotoga maritima class I HK853 with its cognate, RR468, (PDB ID: 3DGE). This interface is used as a template for the spatial complex. Equation (3) is used to calculate energies $H_{TCS}$ and $H_{TCS}^{0}$ at interface positions, where $H_{TCS}$ is calculated using cognate couplings and local fields and $H_{TCS}^{0}$ is calculated using scrambled couplings and local fields. $H_{TCS}^{0}$ is generated using the large-q Potts Hamiltonian model on the scrambled MSA which is constructed by completing 25 rounds of concatenation of any of *m* HKs in the data set with any of *n* RRs in the dataset:

(4) $$\begin{array}{cc} H_{\mathrm{TCS}}^{0}\left(\left\{\mathrm{HK},\mathrm{RR}\right\}\right) & =\\ \langle H_{\mathrm{TCS}}\left({\mathrm{HK}}_{X}\right|X\in \left\{1,\cdots ,m\right\} & +{\mathrm{RR}}_{Y}{|Y\in \left\{1,\cdots ,n\right\})\rangle }_{25} \end{array}$$

To find $H_{\mathrm{TCS}}^{\mathrm{specific}}$, Hamiltonian energies calculated from shared attributes present in all HK–RR pairs must be removed from the specific HK–RR pair being evaluated:

(5) $$\begin{array}{cc} H_{\mathrm{TCS}}^{\mathrm{specific}}\left({\mathrm{HK}}_{A}+{\mathrm{RR}}_{A}\right) & =\\ H_{\mathrm{TCS}}\left({\mathrm{HK}}_{A}+{\mathrm{RR}}_{A}\right) & -H_{\mathrm{TCS}}^{0}\left(\left\{\mathrm{HK},\mathrm{RR}\right\}\right) \end{array}$$

where the resulting $H_{\mathrm{TCS}}^{\mathrm{specific}}$ represents the interaction specificity strength between the HK and RR. Therefore, this energy function could be used to predict the interaction preference between any HK and RR. Additionally, an updated $H_{\mathrm{TCS}}^{\mathrm{specific}}$ score, after incorporating a mutation in the MSA, serves a reference for the effect of the mutation on binding specificity strength. The updated $H_{\mathrm{TCS}}^{\mathrm{specific}}$ is generated by performing the same calculations presented in Equations (3) and (5). Ranges for $H_{\mathrm{TCS}}^{\mathrm{specific}}$ values are varied between organisms and strains where a positive score indicates a loss of shared encoded TCS attributes, a negative score indicates encoded specificity, and a score of zero indicates a presence of all shared TCS attributes but diminished encoded specificity. When qualifying potential interactions, users should compare $H_{\mathrm{TCS}}^{\mathrm{specific}}$ for different TCS pairs belonging to the same organism. One should consider more negative values to have increased encoded specificity, zero values to be capable of interacting with other TCS proteins without encoded specificity in the HisKA domain, and positive values to exhibit insulation of HisKA and REC domains.

### 4.3. Software

The web server has a custom-built front end running React [30] for enhanced user experience with custom components. The back-end is serving data through REST [31] endpoints. Upon mutation, the scores are looked up from a pre-computed table. The python source code for the calculation of $H_{TCS}$ is accessible via the web server. Details on public endpoints can be found in Appendix A.

## 5. Conclusions

The ELIHKSIR web server is a valuable tool for analyzing TCS specificity landscapes in a growing list of 6412 species and strains of bacteria, 65 species and strains of archaea, and 188 species and strains of eukaryotes. This allows users to find potential cross-talk interactions and characterize existing orthogonality for many organisms across different kingdoms. For each organism, heatmaps and histograms of TCS networks are easily accessed, displayed, and exported. Furthermore, the ability to compute, display, and export changes in specificity for mutated HK or RR proteins allows users to explore potential interactions and visualize changes in specificity over an entire signaling network. This ability can assist in the analysis of engineered mutants, clinical and environmental variants, and cross-talk behavior. While ELIHKSIR is useful for interactions between HisKA family HKs and the REC domain of RRs, there exist other HK families in which the ELIHKSIR model does not evaluate. Building and validating models to predict specificity for other families of HK would further assist TCS research. Even though ELIHKSIR only displays specificity scores for HisKA and REC domains, these domains are critical in determining specificity for many TCS interactions, as demonstrated by the 6,272,607 HK-RR pairs evaluated. Due to the ability to mutate each protein and recalculate network-wide specificity scores, there are nearly endless possibilities of HK–RR pairs to evaluate using ELIHKSIR. The accessibility, breadth, and functionality of ELIHKSIR allows a variety of researchers (both computational and experimental) to harness TCS specificity predictions, supporting research efforts through a tool that did not previously exist.

## Figures and Tables

**Table 1:** Attributes of the ELIHKSIR web server.

**Total Organisms**	6676
Bacteria	6412
Archaea	65
Eukaryotes	188
Unknown Organisms/Metagenomes	11
**Total Interactions Evaluated**	6,272,607
Number of HKs	111,032
Number of RRs	225,616

## Data Availability

Data presented in this work is available at the ELIHKSIR web server at https://elihksir.org/.

## Abbreviations

The following abbreviations are used in this manuscript:

ELIHKSIR	Evolutionary Links Inferred for Histidine Kinase Sensors Interacting with Response regulators
TCS	Two-Component System
DCA	Direct-Coupling Analysis
mfDCA	couplings generated by mean-field method as outlined in Morcos, 2011 [1]
DI	Direct Information
HK	Histidine Kinase, Histidine Kinase family (Pfam:PF00512) [15]
RR	Response Regulator, Response Regulator family (Pfam: PF00072) [16]
TP	True Positive
FN	False Negative
PS	Positive Selection
NS	Negative Selection

## Appendix A

Data of the server can be accessed in a programmatic way through two REST endpoints as described in Table A1. The all organisms endpoint api/list returns a list of all the organisms currently accessible through ELIHKSIR. The return value will contain the names (ORGANISM_NAMES::STRING), UNIPROT ID (ORGANISM_UNIPROT_ID::STRING), and the numeric identifier/primary key (ORGANISM_ID::INT) for each organism. By using the numeric identifiers obtained from the list endpoint further meta data and information, along with the scores for each interacting pair, can be obtained through the api/pairs endpoint.

**Table A1:** List of the available endpoints for the REST API.

Endpoint	HTTP Method	URL
All Organisms	GET	api/list
Pairs for heatmap	GET	api/pairs/{ORGANISM_ID::INT}

**Table A2:** Couplings used in specificity model sorted by descending DI value.

HK	RR	DI	HK	RR	DI	HK	RR	DI	HK	RR	DI
18	77	0.102853	7	147	0.00806367	14	170	0.00572271	16	77	0.00460402
22	80	0.0722833	30	83	0.00801695	19	169	0.00571496	42	77	0.00460128
11	167	0.0705232	33	83	0.00781679	14	147	0.00570246	26	176	0.004455
26	84	0.0515243	22	171	0.00767719	22	170	0.00569941	42	76	0.00444185
23	80	0.0492594	19	79	0.00765574	16	76	0.00569788	17	169	0.00442802
14	146	0.04276	23	172	0.00763662	15	76	0.00568733	27	80	0.00440244
46	76	0.0398581	18	171	0.00759788	38	80	0.0056818	15	167	0.00439432
19	76	0.0392644	19	147	0.00753089	10	147	0.00567069	19	81	0.00420284
25	170	0.0351779	18	81	0.00752615	22	172	0.00562105	38	79	0.00418686
25	171	0.0303173	12	148	0.00734758	13	147	0.00559724	41	79	0.00416339
11	168	0.0285807	10	150	0.00732327	34	87	0.00559491	15	170	0.0041264
15	146	0.0270048	45	76	0.007169	33	84	0.00556154	24	173	0.00403743
29	87	0.0265711	22	76	0.00712089	34	84	0.00552466	18	146	0.00382329
19	77	0.0259669	21	172	0.00705718	25	169	0.00545037	16	147	0.00378105
30	87	0.0215653	30	80	0.00702085	15	118	0.00544512	18	172	0.00377972
23	76	0.0193616	15	74	0.00697282	25	174	0.00543429	20	168	0.00375797
19	80	0.0189693	18	147	0.00691656	18	74	0.00541696	16	169	0.00366294
22	77	0.0188355	45	79	0.00690392	14	149	0.00540391	25	81	0.00362641
23	79	0.0180874	22	78	0.00680398	30	86	0.00538702	13	169	0.00359782
19	74	0.0176283	19	170	0.00679327	33	87	0.00538074	10	148	0.00359015
8	147	0.0172729	23	170	0.00679065	17	147	0.00537632	20	77	0.00355346
18	169	0.0171606	18	78	0.00676363	33	86	0.00534217	11	146	0.00345648
29	171	0.0170736	26	81	0.00675062	7	149	0.00530426	21	81	0.00344168
16	168	0.0168674	31	87	0.0067342	14	169	0.00530394	42	80	0.00338203
15	77	0.0152404	21	77	0.00670382	38	83	0.00526882	28	173	0.00334077
25	172	0.0149784	27	84	0.00667465	26	82	0.00525117	22	174	0.0032904
39	83	0.014901	22	81	0.00666247	17	77	0.00524036	20	170	0.00327778
29	172	0.0146187	46	77	0.00658625	42	83	0.00521168	14	168	0.0032269
21	170	0.014469	26	79	0.00657677	34	83	0.00520958	22	176	0.00319442
26	80	0.0141692	18	168	0.00651457	34	80	0.00518935	24	172	0.00310293
26	83	0.0139579	45	80	0.00648245	20	80	0.00514101	17	170	0.00307046
23	83	0.0130196	19	172	0.00639985	46	80	0.00512939	19	168	0.00298315
12	168	0.0128269	18	76	0.00633046	18	170	0.00510377	26	85	0.00290492
15	147	0.0123301	28	172	0.00626959	23	82	0.00509604	20	171	0.0027667
29	84	0.0121863	25	175	0.00623035	25	80	0.00502442	15	169	0.00275228
8	148	0.0119021	16	74	0.00615123	49	77	0.00502117	20	169	0.00269252
23	84	0.0118386	30	88	0.00614559	45	77	0.00501361	15	168	0.00266174
22	84	0.0113964	19	75	0.00610985	24	171	0.00496918	21	168	0.00254422
23	171	0.0108972	10	149	0.00608531	17	76	0.00494564	26	174	0.00238278
32	87	0.0108637	24	80	0.00607124	14	167	0.00492586	27	173	0.00238187
26	171	0.0107505	23	169	0.00605014	15	148	0.00492344	24	169	0.00237797
25	84	0.0104978	33	88	0.00604033	23	173	0.00489615	20	172	0.00233119
14	148	0.0104055	7	150	0.00599047	24	170	0.00488941	18	145	0.00222389
30	84	0.0102802	48	76	0.00596518	44	76	0.00484498	13	168	0.00190674
26	87	0.0102777	21	80	0.0059512	21	173	0.00483877	10	169	0.00186603
23	78	0.01007	25	173	0.00592837	29	173	0.00481891	18	167	0.00162171
23	77	0.00997545	28	171	0.00591566	10	168	0.00480323	12	169	0.00147153
22	169	0.00996165	42	79	0.00586154	17	171	0.00478502	15	145	0.00135628
43	80	0.00972282	22	173	0.00585409	22	168	0.00478308	11	149	0.00105631
22	83	0.00952424	26	172	0.0058532	12	147	0.0047804	11	169	0.000969333
18	80	0.0093605	49	76	0.005838	26	173	0.00476983	11	150	0.000862052
7	148	0.00897782	26	175	0.00581626	22	82	0.00476307	11	148	0.000856593
29	83	0.00882006	30	172	0.00577961	45	75	0.00475623	11	118	0.000790599
21	171	0.00865425	22	79	0.00577265	39	80	0.00473036	11	147	0.000413171
26	170	0.00854487	15	73	0.00576882	27	172	0.00472585			
19	171	0.00831504	41	80	0.00576018	41	76	0.00472158			
17	168	0.008266	30	173	0.00574398	27	83	0.0046522			
19	78	0.00818936	23	81	0.00573789	20	76	0.00465154			
21	169	0.0080992	22	175	0.00573107	16	77	0.00460402			
